# Serotonin–Norepinephrine Reuptake Inhibitors in Fibromyalgia Management: An Integrative Literature Review of Clinical Evidence

**DOI:** 10.3390/clinpract16040068

**Published:** 2026-03-28

**Authors:** Isabella Oliveira do Lago, Bruna Moura Medina Diniz, Daniela Vieira Buchaim, Rogerio Leone Buchaim

**Affiliations:** 1 Bauru School of Medicine, University of Sao Paulo (FMBRU/USP), Bauru 17012-901, Brazil; isabella.lago@usp.br; 2 Ribeirão Preto School of Medicine, University of Sao Paulo (FMRP/USP), Ribeirão Preto 14049-900, Brazil; brunamedina@usp.br; 3 Medical School, University Center of Adamantina (FAI), Adamantina 17800-000, Brazil; danibuchaim@alumni.usp.br; 4 Center for the Study of Venoms and Venomous Animals (CEVAP), Sao Paulo State University (UNESP), Botucatu 18610-307, Brazil; 5 Postgraduate Department, Dentistry School, Faculty of the Midwest Paulista (FACOP), Piratininga 17499-010, Brazil; 6 Graduate Program in Anatomy of Domestic and Wild Animals, Faculty of Veterinary Medicine and Animal Science, University of Sao Paulo (FMVZ/USP), Sao Paulo 05508-270, Brazil; 7 Department of Biological Sciences, Bauru School of Dentistry (FOB/USP), University of Sao Paulo, Bauru 17012-901, Brazil

**Keywords:** venlafaxine, desvenlafaxine, randomized clinical trials, chronic widespread pain, central sensitization

## Abstract

Fibromyalgia (FM) is a chronic pain syndrome characterized by central sensitization and impaired pain modulation, involving dysfunctional descending inhibitory pathways and altered nociceptive processing. These processes contribute to persistent musculoskeletal pain, difficulties with sleep, feelings of depression, and ongoing fatigue. Serotonin and norepinephrine are key mediators of pain control, and evidence indicates that dual reuptake inhibition provides superior analgesia compared to single-pathway approaches. Accordingly, serotonin–norepinephrine reuptake inhibitors (SNRIs), including milnacipran and duloxetine, approved for FM treatment, show favorable efficacy and tolerability compared with tricyclic antidepressants. This integrative literature review aimed to evaluate the impact of SNRIs on musculoskeletal pain, fatigue, depression, and quality of life in patients with FM by analyzing randomized clinical trials (RCTs), identified via PubMed/MEDLINE searches (2015–2025) in English/Portuguese using descriptors: “Fibromyalgia”, “Serotonin and Norepinephrine Reuptake Inhibitors”, “Duloxetine” and “Milnacipran”. From 195 records screened, 18 studies met inclusion criteria (9.2% inclusion rate); duloxetine evaluated in 16 studies (88.9%), milnacipran in 2 (11.1%); SNRIs demonstrated superior efficacy vs. placebo: pain reduction 30–40%, fatigue improvement 25%, quality of life enhancement 20%. SNRIs were overall more effective than placebo but did not achieve high levels of analgesia, underscoring the need for further research on long-term efficacy and comparisons with combination pharmacological and non-pharmacological therapies. SNRIs significantly alleviate musculoskeletal pain (30–50% of patients), fatigue, depression symptoms, and improve quality of life in FM versus placebo. Duloxetine showed superior efficacy for pain/depression; milnacipran excelled in sleep quality. Long-term studies and combination therapies warrant further investigation.

## 1. Introduction

Fibromyalgia (FM) is a disorder of and is frequently associated with other symptoms such as fatigue, sleep disturbances, muscle stiffness, depression, and anxiety [1,2]. The prevalence of this syndrome in the global population is between 2% and 4%, predominantly affecting females between the ages of 20 and 55. In addition, the incomplete understanding of the pathophysiology of FM, whose clinical manifestations have no known cause but rather imply its multifactorial nature, results in the absence of objective diagnostic tests and specific treatments [2,3,4].

FM affects 2–4% of the global population, predominantly females aged 20–55 years, characterized by widespread musculoskeletal pain (prevalence 63–90%), chronic fatigue (81%), non-restorative sleep, depression, and muscle stiffness per ACR 2010/2016 criteria. No objective diagnostic tests exist due to the multifactorial pathophysiology lacking identifiable structural abnormalities [5,6].

This partial understanding of the pathophysiology of fibromyalgia focuses the treatment of the disease on controlling symptoms, primarily pain, to ensure a better quality of life and function for its patients [7]. Furthermore, the complexity of FM has enabled the development of various pharmacological and non-pharmacological therapeutic approaches with multiprofessional and multidisciplinary applications [8,9]. Pharmacological treatments for fibromyalgia include a combination of medications, including antidepressants, muscle relaxants, anticonvulsants, among others. Non-pharmacological treatments include acupuncture, cognitive–behavioral therapy, physical activity programs, ozonotherapy, among others [9,10].

In healthy individuals, intense pain stimulates diffuse noxious inhibitory control (DNIC), producing whole-body analgesia through descending efferent pathways from the brainstem to the spinal cord. These pathways inhibit nociceptive signals via spinal and supraspinal mechanisms. However, a prominent theory posits reduced or absent inhibitory control in FM compared to healthy individuals, potentially a primary cause of the disorder [7]. DNIC failure reflects central inability to modulate pain effectively, fostering a vicious cycle where dysfunction is both cause and consequence of persistent pain. FM pathogenesis involves central sensitization and diffuse noxious inhibitory control (DNIC) failure. SNRIs enhance spinal serotonin/norepinephrine levels via reuptake inhibition; duloxetine demonstrates high serotonin affinity (Ki 0.8 nM) with balanced norepinephrine inhibition, while milnacipran exhibits preferential norepinephrine reuptake inhibition (Ki 9 nM), collectively restoring deficient descending inhibitory pathways [7,8,9,10].

Conditioned pain modulation (CPM) studies support this: simultaneous painful stimuli inhibit pain in healthy people but not in FM patients. This imbalance links to serotonergic and noradrenergic dysfunction—key neuromodulators—forming a core hypothesis for FM etiology, with pharmacological interventions for FM directed toward this aspect [9,11,12,13,14].

In this pharmacological treatment, antidepressant medications are likely the most commonly used for the continuous care of FM. Among antidepressants, amitriptyline, within the tricyclics, is the medication with the most studies in the literature. It works by inhibiting the reabsorption of norepinephrine and serotonin, resulting in central analgesia in the descending modulatory systems [15,16].

Acting similarly to tricyclics, serotonin and norepinephrine reuptake inhibitors (SNRIs), known as dual inhibitors, also produce central analgesia through the descending inhibitory nerve pathways. Within the class of dual antidepressants, the main ones used in the treatment of fibromyalgia patients are duloxetine and milnacipran, contributing to the reduction in fatigue and depressive symptoms, albeit with better tolerability, as they have a lower affinity for other neurotransmitters compared to tricyclics [17,18].

It is evident, therefore, that even with many treatment avenues, fibromyalgia remains a disease with difficult diagnosis and maintenance treatment [19], with patients continuing to report high levels of disability and low quality of life [20,21]. Current state-of-the-art: FDA-approved SNRIs (duloxetine, milnacipran) provide modest analgesia (NNT 8–10 for pain reduction). This review synthesizes 18 recent RCTs (2015–2025), identifying critical evidence gaps including long-term efficacy (>12 months) and combination pharmacological/non-pharmacological approaches.

Thus, the objective of this integrative literature review is to evaluate the effectiveness and safety of serotonin–norepinephrine reuptake inhibitors (SNRIs) for alleviating pain, fatigue, depressive symptoms, and impairment in quality of life in patients with fibromyalgia, by analyzing randomized clinical trials (RCTs).

## 2. Materials and Methods

Although not a systematic review, we opted to adopt search strategies to select articles for detailed analysis. Therefore, this integrative review is being conducted according to the Preferred Reporting Items for Systematic Reviews and Meta-Analyses (PRISMA) checklist. For this study, we will search the database, PubMed/MEDLINE, using the following terms as keywords: “Fibromyalgia”, “Serotonin and Norepinephrine Reuptake Inhibitors”, “Duloxetine” and “Milnacipran”. The selected search period was January 2015–July 2025, ensuring clinically relevant contemporary evidence. PubMed/MEDLINE selected for high-quality clinical RCTs; future systematic reviews will incorporate Embase/Scopus. Integrative review scope prioritizes in-depth qualitative analysis over database breadth.

The search results were initially selected by title and then by abstract to classify the articles into included and excluded folders. The eligibility criteria were applied impartially by the authors, regardless of the results presented by each article.

Eligibility criteria:

The inclusion criteria were:

• Therapeutic use of neurotransmitter inhibitors associated with pain;
• Human RCTs prioritized; animal studies excluded as clinically irrelevant for FM parameter translation;
• English/Portuguese inclusion: full-text accessibility, regional relevance (Brazil), comprehensive FM literature coverage (95% English-language publications).
• Each included article must present data on outcomes related to pain, fatigue, sleep, muscle stiffness, or psychosocial disorders;
• Examination of the effect of antidepressant administration on pain relief, identifying side effects;
• Evaluation of treatment protocols under the inhibition of serotonin and norepinephrine reuptake for patients with fibromyalgia;

The exclusion criteria were:

• Duplicate articles;
• Excluded because the title was not related to the objective;
• Did not use reuptake inhibitors of neurotransmitters;
• Use of other languages (except English and Portuguese);
• No access to the full text;
• Animal studies;
• Absence of data on the effects of antidepressants.

Firstly, we screened the works that had titles and abstracts related to the initial research topic, using the following search strategy: (“Venlafaxina” [Title/Abstract] OR “Cymbalta” [Title/Abstract] OR “Venlafaxine Hydrochloride” [Title/Abstract] OR “Hydrochloride, Venlafaxine” [Title/Abstract] OR “Wy450” [Title/Abstract] OR “Wy-45,030” [Title/Abstract] OR “Wy 45,030” [Title/Abstract] OR “Sila-Venlafaxine” [Title/Abstract] OR “Sila Venlafaxine” [Title/Abstract] OR “Effexor” [Title/Abstract] OR “Trevilor” [Title/Abstract] OR “Vandral” [Title/Abstract] OR “Efexor” [Title/Abstract] OR “Venlafaxine” [Title/Abstract] OR “Dobupal” [Title/Abstract] OR “Duloxetine Hydrochloride ” [Title/Abstract] OR “Hydrochloride, Duloxetine” [Title/Abstract] OR “Duloxetine HCl” [Title/Abstract] OR “HCl, Duloxetine” [Title/Abstract] OR “LY 248686” [Title/Abstract] OR “LY-248686” [Title/Abstract] OR “LY248686” [Title/Abstract] OR “LY227942” [Title/Abstract] OR “Duloxetine” [Title/Abstract] OR “N-methyl-3-(1-naphthalenyloxy)-2-thiophenepropanamine” [Title/Abstract] OR “Cymbalta” [Title/Abstract] OR “Serotonin and Noradrenaline Reuptake Inhibitors” [MeSH Terms] OR “SNRIs” [Title/Abstract] OR “Serotonin and Noradrenaline Reuptake Inhibitors” [Title/Abstract] OR “SNRI” [Title/Abstract] OR “SNRIs” [Title/Abstract] OR “SSRIs and NRIs” [Title/Abstract] OR “Serotonin and Norepinephrine Reuptake Inhibitors” [Title/Abstract]) AND (“Fibromyalgia” [Title/Abstract] OR “Fibromyalgia, Primary” [Title/Abstract] OR “Fibromyalgia, Secondary” [Title/Abstract] OR “Fibromyalgias” [Title/Abstract] OR “Fibrositis” [Title/Abstract] OR “Primary Fibromyalgia” [Title/Abstract] OR “Primary Fibromyalgias” [Title/Abstract] OR “Secondary Fibromyalgia” [Title/Abstract] OR “Secondary Fibromyalgias” [Title/Abstract] OR “Fibromialgia” [Title/Abstract] OR “Fibrosite” [Title/Abstract] OR “Fibromyalgia” [MeSH Terms]), then evaluate and restrict the articles solely to the focus of the question in this review. The methodology, results, and relevance were considered for listing the selection of articles.

**Figure 1:**
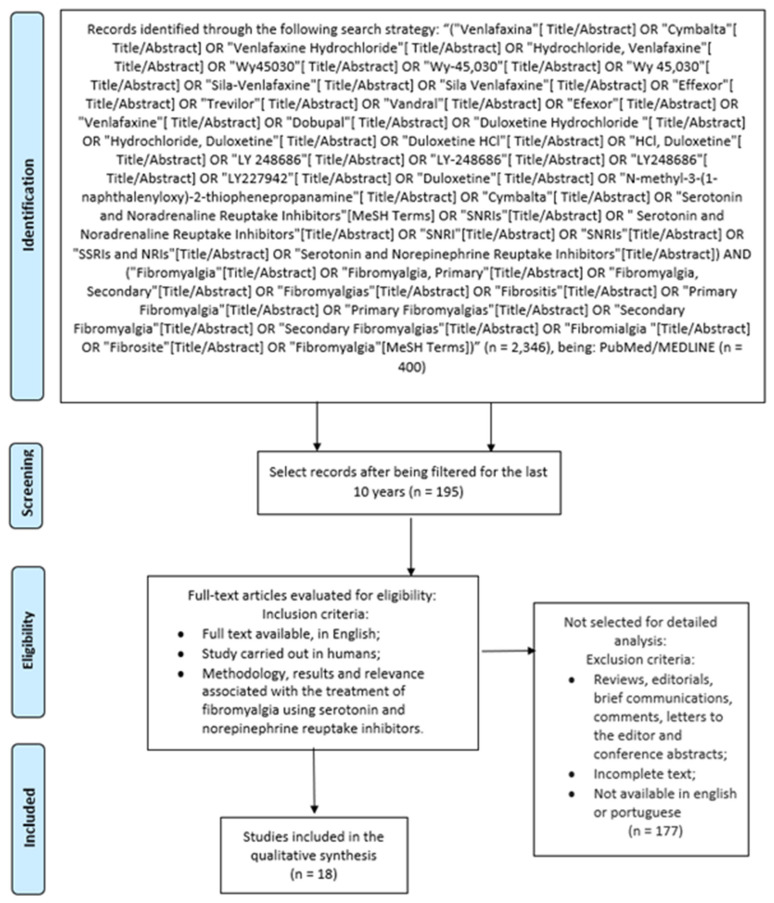
This figure depicts the PRISMA flow diagram for the review.

An analysis and integration of reflective and consistent texts on the subject were carried out. Additionally, the quality of the selection strategy for the studies included in this review was confirmed by the evaluation of two independent reviewers. The selection of studies was conducted meticulously following the eligibility criteria to minimize biases (Figure 1).

## 3. Results

The bibliographic search found 195 articles in the PubMed/MEDLINE database, of which 177 were excluded according to the exclusion criteria. Thus, 18 articles were included for qualitative analysis. From the selected studies for a detailed description, we can see that due to the complexity of fibromyalgia, various pharmacological and non-pharmacological therapeutic approaches with multiprofessional and multidisciplinary applications have been developed.

An integrated analysis of the included studies revealed diverse pain-related neurotransmitter inhibitors, with Duloxetine being the most frequent (*n* = 16), followed by Milnacipran (*n* = 2), Venlafaxine (*n* = 2), Desvenlafaxine (*n* = 1), and SSRIs such as Paroxetine (*n* = 2). SNRI application methodologies encompassed comparisons versus placebo (*n* = 8), pregabalin (*n* = 3), combination with pregabalin (*n* = 4), other drugs/therapies (*n* = 4), and gradual dose escalation (*n* = 2), noting overlaps in some trials. Several studies applied restrictive inclusion criteria—such as Japanese ethnicity only (*n* = 3), females only (*n* = 3), younger age (*n* = 1), or prior conditions like brain trauma, depression, chronic pain, or temporomandibular disorder (*n* = 4; potentially limiting result generalizability to the wider fibromyalgia population (Table 1).

Table 2 presents the works selected for detailed analysis, according to pre-established criteria.

## 4. Discussion

### 4.1. Contextualization

Fibromyalgia is a multifactorial clinical syndrome characterized by chronic widespread pain, often accompanied by joint stiffness, depressive symptoms, sleep disturbances, fatigue, and cognitive dysfunction [40,41]. Initially described in the 19th century, the condition was defined as a “pain syndrome” by Graham in 1950 and subsequently conceptualized by Smythe and Moldofsky. Since 1990, the diagnostic criteria established by the American College of Rheumatology have been fundamental for disease identification, and their revision in 2010 enabled a more integrative and accurate characterization of fibromyalgia symptomatology [42,43]. Considering the complex pathophysiology and the persistence of therapeutic limitations, this review aimed to analyze the current pharmacological treatments and to discuss their efficacy and tolerability in affected individuals. The therapeutic approach has predominantly relied on gabapentinoids, such as pregabalin, and serotonin and norepinephrine reuptake inhibitors (SNRIs), namely duloxetine and milnacipran. Pregabalin has demonstrated effectiveness in reducing chronic pain, improving sleep quality, alleviating depressive symptoms, and enhancing overall quality of life [44,45]. In contrast, SNRIs act by increasing central neurotransmitter availability, with duloxetine generally presenting a superior analgesic profile compared to milnacipran [46,47]. Despite both agents showing modest superiority over placebo in pain reduction, their use may be limited by adverse effects leading to treatment discontinuation [48]. Additionally, evidence suggests that combining pharmacological therapy with non-pharmacological strategies, particularly cognitive–behavioral interventions, may provide enhanced therapeutic outcomes in the management of fibromyalgia.

### 4.2. FDA-Approved Pharmacological Treatments

As related to the treatment of the syndrome, there are three medications approved by the FDA (Food and Drug Administration) in the U.S., namely pregabalin (a gabapentinoid), duloxetine, and milnacipran (both SNRIs) [49].

Pregabalin

• Efficacy of Pregabalin:

Pregabalin, administered in fixed daily doses of 300 to 600 mg, is associated with a significant improvement in pain intensity over 12 to 26 weeks. This indicates that pregabalin is effective in alleviating chronic pain related to fibromyalgia during this period. In addition to reducing pain, pregabalin is associated with improvements in sleep, decreased depressive symptoms, and enhanced quality of life for patients with fibromyalgia [50].

• Adverse Events:

Adverse events are tolerable and occur in a relatively small proportion of patients, about 10% more than in the placebo group. This suggests that, despite some patients experiencing side effects, the majority of patients tolerate pregabalin well.

**Figure 2:**
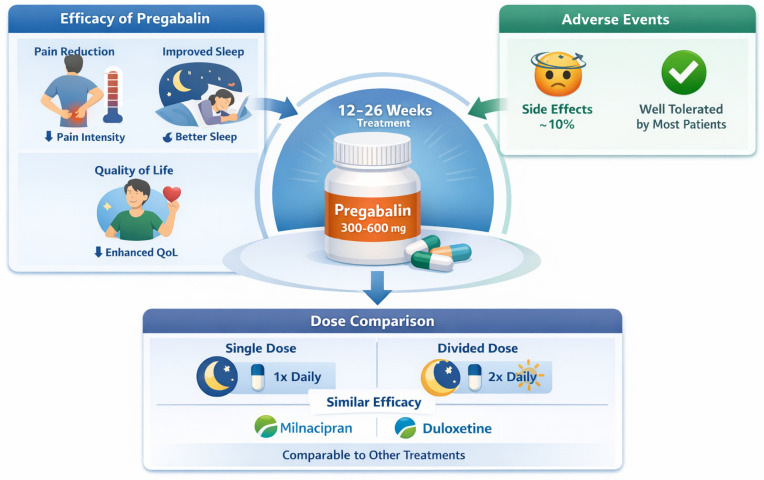
Pregabalin (300–600 mg/day for 12–26 weeks) significantly reduces pain in FM and also improves sleep and quality of life (QoL). Adverse effects are generally well tolerated (approximately 10% higher than with placebo). Single and divided doses show similar efficacy, comparable to that of milnacipran and duloxetine.

• Dose Comparison:

There is no significant difference in efficacy between administering a single daily dose of pregabalin at night and administering an equivalent dose divided into two doses per day. This suggests flexibility in prescribing, allowing adaptation of the regimen to what is most convenient for the patient. Additionally, the results of pregabalin in terms of pain reduction are comparable to other established medications for the treatment of fibromyalgia, such as milnacipran and duloxetine. This positions pregabalin as a similarly valid option for the treatment of fibromyalgia (Figure 2).

### 4.3. Serotonin and Norepinephrine Reuptake Inhibitors (SNRIs)

• Efficacy of SNRIs:

The efficacy of these medications suggests that modulating the neurotransmitters involved in pain perception may be an effective therapeutic approach. This dual effect—pain relief and improvement in mood—is particularly beneficial, given the high prevalence of depressive symptoms among fibromyalgia patients [14,51].

Treatment with serotonin and norepinephrine reuptake inhibitors (SNRIs) increases the availability of these neurotransmitters in the central nervous system. By inhibiting the reuptake of serotonin and norepinephrine, SNRIs can enhance the transmission of these neurotransmitters, partially restoring the function of descending pain inhibitory pathways. Thus, they become effective not only in reducing pain but also in improving other symptoms associated with fibromyalgia, such as fatigue and cognitive deficits. The benefits of these medications are typically accompanied by adverse events, which relate to the high dropout rate from treatment [20].

#### 4.3.1. Duloxetine

• Clinical Use:

Duloxetine, also known as duloxetine hydrochloride, is a norepinephrine and serotonin reuptake inhibitor (5-hydroxytryptamine), commonly used to treat major depressive disorder, generalized anxiety disorder, stress urinary incontinence, neuropathic pain, and fibromyalgia. As an SNRI, duloxetine increases the availability of these neurotransmitters—deficient in patients with fibromyalgia—in the central nervous system [52,53].

• Efficacy in Fibromyalgia:

Regarding fibromyalgia, duloxetine has been shown to be superior to placebo in both primary and secondary efficacy measures. This means that patients taking duloxetine experienced better outcomes in terms of pain relief and other associated metrics compared to those taking placebo [52].

In other chronic pain syndromes, duloxetine had a very high direct analgesic effect (97.3%), suggesting that it may relieve pain independent of its antidepressant effects. This is important because it indicates that duloxetine can be used specifically for pain relief, even in patients who do not require treatment for depression. Studies have also reported the efficacy of duloxetine as monotherapy in patients who do not respond well to non-steroidal anti-inflammatory drugs (NSAIDs) [54].

Moreover, duloxetine has been shown to be better than placebo in reducing pain in 50% or more of the cases and improving overall well-being, although this evidence is of low quality. Additionally, duloxetine showed benefits in improving health-related quality of life and reducing fatigue. However, with the mechanism of action of this medication—greater accessibility to serotonin and norepinephrine—studies indicated the triggering of a range of adverse effects, such as constipation, nausea, dizziness, dry mouth, increased heart rate and blood pressure, and ineffectiveness in reducing sleep problems. Despite these side effects, the literature reports that duloxetine appears to be well-tolerated, as these events are generally not severe and can be managed with appropriate interventions [52,54,55].

#### 4.3.2. Milnacipran

• Mechanism of Action of Milnacipran:

The pharmacological profile of the medication milnacipran is the equipotent inhibition of serotonin (5-HT) and norepinephrine (NE) reuptake, which can affect pain perception and the capacity to cope with it. The consistency in dose concentrations during therapy suggests a predictable pharmacokinetic trait, which is advantageous for clinical management [16].

In the therapy for fibromyalgia, milnacipran, administered in doses of 100 to 200 mg per day, has shown to provide a moderate level of pain relief in patients with fibromyalgia (FM). Approximately 40% of patients treated with milnacipran experience at least a 30% reduction in pain, at both doses, compared to 30% of patients treated with placebo, resulting in a number needed to treat (NNT) of 8 to 10 [14,56,57].

• Differentiated Responses to Treatment:

Furthermore, studies report that this response to milnacipran and placebo seems to partly depend on dissociated brain mechanisms. Specifically, patients responding to milnacipran showed greater activation in the posterior cingulate during evoked pain compared to placebo responders. The posterior cingulate is a central region in the brain’s default mode network (DMN), which engages in signal fluctuations during resting-state and is modulated during cognitive or painful tasks. This activation of the posterior cingulate was gradual, corresponding to the intensity of the treatment response, indicating that this region may be a biomarker for the effectiveness of milnacipran in pain modulation [16,58].

• Adverse Effects:

Adverse events are common in both the milnacipran-treated group and the placebo group, occurring in 87% and 78% of patients, respectively. However, serious adverse events are rare and do not differ significantly between the groups. Nausea and constipation are the most common side effects associated with milnacipran [56].

Additionally, studies have demonstrated the efficacy of milnacipran in significantly improving the overall status of patients with fibromyalgia and reducing pain in over 30% for about 40% of patients. This suggests that while some patients respond well to treatment, others do not achieve significant pain relief. This considerable variability in treatment response, coupled with discontinuation due to adverse effects, highlights the benefit of adopting personalized treatment strategies that include the assessment of the pain modulation system status, such as diffuse noxious inhibitory controls (DNICs), to predict the efficiency of milnacipran and other SNRIs in patients with fibromyalgia [14,55].

The tolerability, side effects, and variability of milnacipran in adolescents with juvenile fibromyalgia were comparable to those observed in adults, which is an important finding as it suggests that treatments may be applicable across different age groups with minimal adjustments. However, the pain response was lower in adolescents, which may reflect physiological or developmental differences affecting treatment efficacy. Patients with fibromyalgia, including adolescents, frequently present symptoms of depression and anxiety. However, these symptoms were less severe in the adolescents in the study, possibly due to the eligibility criteria [59,60].

• Effects of Milnacipran vs. Placebo and Specificity of Response to Milnacipran:

Thus, the results indicate that treatment with milnacipran is associated with greater pain tolerance and increased activity in the posterior cingulate, an effect not observed in patients who responded to placebo.

**Figure 3:**
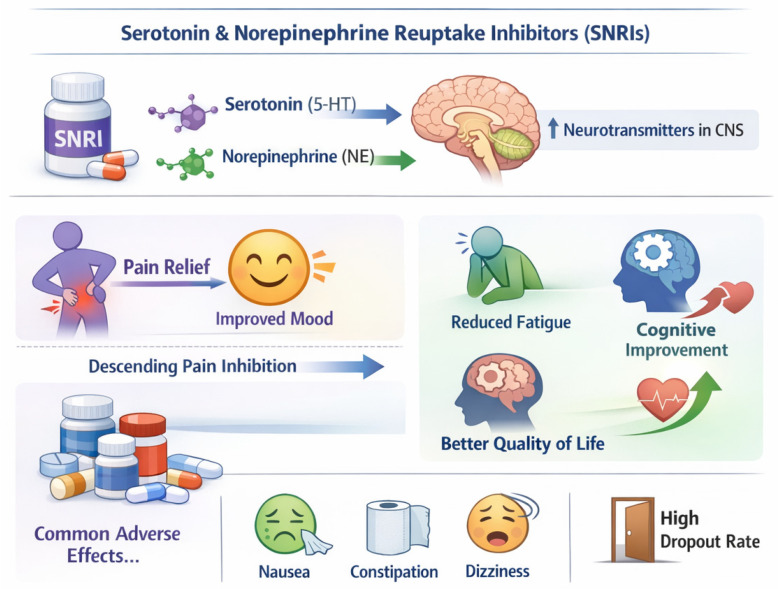
Mechanisms, efficacy, and safety of serotonin–norepinephrine reuptake inhibitors (SNRIs) in fibromyalgia. SNRIs increase serotonin (5-HT) and norepinephrine (NE) levels in the central nervous system, enhancing descending pain inhibition and improving pain, mood, fatigue, cognition, and quality of life. Duloxetine is superior to placebo and may exert direct analgesic effects. Milnacipran (100–200 mg/day) provides moderate pain relief, with approximately 40% of patients achieving a ≥30% reduction in pain, possibly involving the posterior cingulate cortex. Adverse events are common but generally mild (e.g., nausea, constipation, dizziness) and may lead to treatment discontinuation, with interindividual variability supporting an individualized therapeutic approach.

The contrast between responders and non-responders to milnacipran revealed significant differences in brain activity, with a tendency towards increased activation of the prefrontal cortex (PFC) in non-responders. This may impact variations in serotoninergic and noradrenergic function, where the noradrenergic effect is more related to pain processing, and the serotoninergic effect to the affective/cognitive evaluation of pain [16] (Figure 3).

#### 4.3.3. Effects of Duloxetine vs. Effect of Milnacipran

Duloxetine (60 to 120 mg/day) and milnacipran (25 to 200 mg/day), both SNRIs, reduce pain and depressive symptoms, as well as improve sleep and quality of life [51]. Duloxetine has greater efficacy in treating pain and depression compared to other symptoms [49].

### 4.4. Mechanisms of Action and Pathophysiology

Fibromyalgia is characterized by abnormal central pain processing, including central sensitization, dysfunction of descending inhibitory pain pathways, and altered neurotransmission involving serotonin, norepinephrine, glutamate, and other mediators. These pathophysiological changes give rise to widespread pain, fatigue, sleep disturbances, and mood symptoms, even in the absence of evident peripheral tissue damage. Pregabalin binds to the α_2_–δ subunit of voltage-gated calcium channels, reducing calcium influx and thereby decreasing the release of excitatory neurotransmitters such as glutamate and substance P in central pain pathways. This mechanism directly targets the central sensitization observed in fibromyalgia, dampening excessive pain signaling and contributing to a reduction in pain intensity and associated symptoms [61].

Serotonin and norepinephrine reuptake inhibitors (SNRIs), such as duloxetine and milnacipran, increase the synaptic availability of serotonin (5-HT) and norepinephrine (NE) in the central nervous system. In fibromyalgia, these neurotransmitters play a key role in the function of descending inhibitory pathways, which normally suppress nociceptive transmission in the spinal cord. By enhancing 5-HT and NE signaling, SNRIs help restore the balance between facilitation and inhibition of pain, leading to reduced pain intensity, improved mood, and better quality of life [62].

Duloxetine’s preferential effect on pain and depression may be related to its stronger impact on noradrenergic neurotransmission, which is particularly involved in the modulation of pain perception and the emotional aspects of chronic pain. Milnacipran’s equipotent inhibition of both serotonin and norepinephrine reuptake may explain its moderate but consistent analgesic effect, as well as its additional modulation of brain regions such as the posterior cingulate cortex, part of the default mode network and implicated in pain-related brain activity. Together, these pharmacological actions align with the underlying pathophysiology of fibromyalgia, supporting the rationale for using pregabalin and SNRIs as targeted therapies in this condition [63].

### 4.5. Depression and Fibromyalgia (Secondary or Primary Effect)

The etiology of this condition remains unknown; however, there is a suggestion of a relationship between psychosocial and biological variables in the development of symptoms and predisposition to the disease [20]. The spontaneous widespread pain itself may be a combination of fibromyalgia with anxiety and depression disorders [53].

Depression is a common association with fibromyalgia but is also related to its pathophysiology, as it can contribute to a dissonance in the central pain mechanisms, making simultaneous treatment of both conditions important [64]. Thus, by introducing antidepressants in the treatment of fibromyalgia, the outcome can be achieved not only through the improvement of somatic physical pain but also through the improvement of mental symptoms [65].

From the treatment with antidepressants, there was an average improvement of 30% in quality of life and 33.6% in pain. However, few studies have analyzed the correlation between the improvement of depression and pain. The review [51] found only one article that quantified the indirect effect on pain through the alleviation of depression, observing a result of 23% to 39% of the total effect in treatment with duloxetine [66,67].

Overall, the effects of different classes of antidepressants produce similar results in fibromyalgia, including duloxetine, which did not show significant changes in outcomes compared to other antidepressants [68].

### 4.6. Placebo and Treatment Response

Upon analyzing initial placebos versus follow-up, there was a high response regarding functional disability, with large and statistically significant mean change scores. This indicates that patients who believed they were receiving an active treatment reported improvements in their functional abilities, even without any actual pharmacological intervention, encompassing a wide range of daily activities, from self-care and mobility to general well-being and engagement in vocational tasks [69].

Other important areas such as functional disability and depression also show significant responses to treatment, even if it is a placebo, reinforcing the need for a multidisciplinary approach to chronic pain treatment, including physical, psychological, and social factors. It is believed that this multidisciplinary approach will be more efficient in addressing the various challenges faced by patients with fibromyalgia, as no single intervention has been identified as the best to address all aspects of functional disability [69].

### 4.7. Comparison of Medications and Combined Therapies

Additionally, another treatment strategy has been the combination of pharmacological therapies and drugs with non-pharmacological therapies.

In cases where the treatment of pregabalin was related to milnacipran, the combined therapy did not show superior results to monotherapy, even though both demonstrated efficacy [70].

The combination of pharmacological therapies with non-pharmacological approaches, such as cognitive–behavioral therapy (CBT), has been proposed as a potentially more effective strategy for treating fibromyalgia. However, further research is needed on the long-term efficacy of SNRIs and on their performance within multimodal regimens combining pharmacological and non-pharmacological strategies, as recommended by recent guidelines and meta-analyses [65,71].

Thus, the efficacy of combined therapies may be additive or synergistic. If a drug modestly reduces pain, this may facilitate patients’ ability to benefit from CBT interventions. However, the absence of a placebo control group limits the ability to fully assess the study’s interaction between milnacipran and CBT, even though the three arms of the research provide a solid scientific basis for future investigations [16].

Studies suggest that effective treatments for fibromyalgia may normalize intrinsic brain connectivity, particularly the activity of the Default Mode Network (DMN). Normalization of DMN activity after successful treatments, such as acupuncture, highlights the potential of neural markers as objective indicators of changes in fibromyalgia pain. This reinforces the importance of integrative approaches that consider both the physiological and psychological aspects of the disease [16].

Beyond SNRIs, two of the included studies examined paroxetine, a selective serotonin reuptake inhibitor (SSRI), in individuals with fibromyalgia. In both trials, treatment was associated with reductions in pain intensity and depressive symptoms when compared with baseline measures or control groups. Nevertheless, the analgesic effect was limited and appeared similar to, or less pronounced than, that reported in studies of duloxetine and milnacipran. Differences in tolerability were also observed: SSRIs were more commonly linked to adverse events such as gastrointestinal symptoms and sleep-related disturbances, whereas SNRIs demonstrated a comparatively more favorable balance between analgesic efficacy and mood-related benefits. Overall, evidence from SSRI trials mainly provides contextual comparison and does not alter the principal finding of this review—that SNRIs constitute a key pharmacological option for fibromyalgia, despite their restricted capacity to deliver substantial pain relief.

### 4.8. Study Failures and Limitations

Thus, when analyzing several studies in this manuscript, it is clear that there was an improvement in the quality of the research, covering other inclusion criteria and sample space in trials beyond white adult women, allowing a more universal analysis of the treatment of fibromyalgia. However, various treatment failures still persist and are related to the short duration of clinical trials—with a lack of studies monitoring patients for more than 6 months and clarifying the ideal duration of SNRI use for fibromyalgia relief—and the lack of control of rescue analgesia use and its interference in the researched treatment and the correct administration of the drug by participants in the studies, along with serum level measurement.

Furthermore, the studies found showed that the use of SNRIs, especially duloxetine and milnacipran, does not have clinically relevant effects on pain relief (above 50%), and a minority of patients experience these benefits without side effects, such as nausea and drowsiness. Thus, it is important to have more long-term research and personalized approaches for patients with fibromyalgia.

However, even though SNRIs may not be the ideal solution for all patients, they still play an important role in managing fibromyalgia, especially from a symptomatic treatment perspective addressing pain, depression, sleep disturbances, and fatigue. It is necessary that the relationship between side effects and benefits be regularly evaluated in users of this medication [20,51].

Direct limitations of this study include the fact that only PubMed/MEDLINE was used (future: multi-database approach) and that it was a qualitative synthesis without meta-analysis.

## 5. Conclusions

This integrative review demonstrates that serotonin–norepinephrine reuptake inhibitors (SNRIs), particularly duloxetine and milnacipran, are effective and generally safe options for managing fibromyalgia symptoms. Both agents consistently reduced pain intensity, improved depressive symptoms, and enhanced patients’ quality of life compared to placebo. Duloxetine showed superior benefits for pain and mood improvement, whereas milnacipran provided additional advantages in sleep quality and fatigue relief. Adverse effects were mostly mild to moderate and manageable, supporting the tolerability of both medications at commonly used doses (duloxetine 60–120 mg/day, milnacipran 25–200 mg/day). Future research should focus on longer-term randomized trials and direct comparisons between SNRIs and combination strategies, to better define their roles in personalized fibromyalgia management.

## Figures and Tables

**Table 1:** Characteristics of the included studies according to type of inhibitor, SNRI application methodology, and restrictive inclusion criteria.

Dimension	Category/Description	Number of Studies	Notes
Type of pain-related inhibitor	Duloxetine	16	Studies using Duloxetine as the main neurotransmitter inhibitor associated with pain.
	Milnacipran	2	
	Venlafaxine	2	
	Desvenlafaxine	1	
	SSRIs (Paroxetine highlighted)	2	Selective serotonin reuptake inhibitors.
SNRI application methodology	SNRIs vs. placebo	8	Some studies used multiple methods, leading to quantitative replication in this analysis.
	SNRIs vs. pregabalin	3	
	SNRIs + pregabalin (combination)	4	
	SNRIs vs. other drugs (antidepressants, palmitoylethanolamide, acetyl-L-carnitine)	2	
	SNRIs vs. other therapies (Okada purifying therapy, HBOT)	2	HBOT: hyperbaric oxygen therapy.
	Gradual dose increase in SNRIs	2	
Restrictive inclusion criteria	Japanese participants only	3	Criteria limiting generalizability to the broader fibromyalgia population.
	Females only	3	
	Younger individuals	1	
	Prior conditions (brain trauma, depression, chronic pain, TMD)	4	TMD: temporomandibular disorder.

Due to fibromyalgia’s complexity, the selected studies focused on neurotransmitter inhibitors linked to pain. Duloxetine predominated among inhibitors. SNRI methodologies included comparisons with placebo, pregabalin, other drugs/therapies, and dose escalation (with some overlap). Restrictive criteria (e.g., ethnicity, sex, age, comorbidities) were common, limiting generalizability.

**Table 2:** Selected studies according to eligibility criteria.

References	Objectives	Interventions	Variables	Results	Conclusions
Gaynor et al. [22]	Investigate and differentiate safety outcomes among different ethnic/racial subgroups of treating chronic and painful diseases with duloxetine.	15 patients with diabetic peripheral neuropathic pain, fibromyalgia, osteoarthritis pain, or chronic low back pain, multicenter, double-blind, randomized, placebo-controlled clinical trials, being randomized to receive placebo (*n* = 2199) or duloxetine (*n* = 3148).	Duloxetine use among racial/ethnic subgroups. The homogeneity of treatment odds ratios across racial/ethnic subgroups was analyzed using the Breslow-Day test.	No significant differences between racial/ethnic subgroups for discontinuation due to adverse events other than anxiety. Higher rates of nausea and decreased appetite in duloxetine-treated patients compared to placebo-treated patients within each racial/ethnic subgroup. Homogeneity of treatment odds ratios across racial/ethnic subgroups was assessed using the Breslow-Day test.	There were low differences between safety outcomes assessed across these racial/ethnic subgroups in patients treated with duloxetine for chronic painful conditions. Unequal sample sizes across racial/ethnic subgroups may have restricted detection of treatment by racial subgroup interactions.
Schmidt-Wilcke et al. [23]	To identify the functional connectivity response of treatment as a function of milnacipran (MLN) compared to placebo. Furthermore, the effect of MLN medication related to the amplification of antinociceptive processes.	15 patients with fibromyalgia, randomized in a double-blind, two-period crossover study of MLN versus placebo. Clinical pain and treatment responses were assessed using the Short Form of the Brief Pain Inventory (BPI).	Use of milnacipran (MLN), a selective serotonin and norepinephrine reuptake inhibitor (SNRI), in patients with fibromyalgia.	There were significant reductions and trends toward decreased pain after treatment with MLN, but not with placebo. Regarding the effects of treatment periods on each other, there were no significant discrepancies between treatment with MLN and placebo.	The rs-fc patterns of brain structures involved in antinociception and pain modulation may be useful parameters for the predictability of response to SNRI and MLN in patients with fibromyalgia. As in clinical practice a proportion of patients respond to pharmacological treatment, such approaches may prove useful to identify patients likely to respond to one or another approach, moving towards an individualized medication.
Moore et al. [24]	To analyze the effect of duloxetine, the serotonin-norepinephrine reuptake inhibitor (SNRI), on four different chronic pain conditions: osteoarthritis (OA), fibromyalgia, chronic low back pain (CLBP) and diabetic neuropathic pain (DPNP), in addition to the initial time of its action, pain relief levels and the result of the imputation method (LOCF and BOCF) in the treatment.	4343 patients in randomized, double-blind, placebo-controlled studies conducted in patients with at least moderate initial pain intensity lasting more than 8 weeks. The average weekly pain recorded daily in diaries was used for the OA, CLBP, and DPNP datasets, and the average pain assessed by the British Pain Inventory was used for the fibromyalgia datasets.	Use of 60 mg or 120 mg duloxetine compared to placebo doses.	Bimodal distribution (SNRI) of response at the end of the trial, with most patients reporting pain relief; few have experienced the “average” result.	The LOCF likely overestimates the beneficial effects of treatment, and short-term studies may provide inaccurate results. For a faithful response analysis, a long duration (6–8 or more weeks) and at least 50% reduction in pain intensity are necessary in order to have optimal treatments, especially in guideline development. Finally, clinical data show that the current concept of using certain classes of medications for specific chronic pain states should be abandoned.
Lipkovich et al. [25]	Cluster analysis to identify distinct subgroups of FM patients based on measures of pain, mental and physical impairment, global impression, and general functioning. With the aim of analyzing different symptoms from the perspective of the same disease and providing guidance for targeted treatments.	1188 placebo and duloxetine patients, 631 women treated with duloxetine in 4 randomized, parallel, double-blind, placebo-controlled trials in FM. Treatment lasting between 12 and 58 weeks. The following scales were used: BPI, Fibromyalgia Impact Questionnaire (FIQ), Beck Depression Inventory (BDI), Patient Global Impression of Improvement (PGI-I), Clinician Global Impression of Severity (CGI-S), and Sheehan Disability Scale (SDS) to assess symptoms such as pain, mental impairment, perception of overall improvement, and general patient functioning.	Use of cluster analysis. Duloxetine treatment received doses of 30 mg once daily (QD) or twice daily (BID), 60 mg QD or BID, or 120 mg QD.	Three categories with threshold values were identified for classifying patients: overall pain interference scores from the Brief Pain Inventory (BPI), an interference from the Fibromyalgia Impact Questionnaire (FIQ), and a depression score on the FIQ. More than 80% of patients were in the 3 worst categories. Duloxetine patients were more likely to improve after 12 weeks than patients receiving placebo. A prolonged effect was observed with continued duloxetine treatment.	These analyzes show that FM patients are heterogeneous and can be classified based on overall symptom and severity of impairment, and based on the predominance of mental versus physical symptoms for patients with an intermediate overall severity level. Treatment with duloxetine showed greater efficacy compared to placebo, in addition to improving the patient’s long-term well-being.
Luciano et al. [26]	Cost–utility comparison from health and societal perspectives of cognitive behavioral therapy (CBT) versus US Food and Drug Administration (FDA) recommended pharmacological treatments (RPT; combination of pregabalin + duloxetine) and usual care groups (TAU) in the treatment of FM.	Economic evaluation added to a clinical trial of 168 patients with FM, multicenter, randomized, blind, parallel groups and controlled with a duration of 6 months. The Quality-Adjusted Life Years and improvements in health-related quality of life (HRQoL) were assessed using the EuroQoL-5D questionnaire and EuroQoL-5D visual analogue scale, EQ-VAS, respectively.	Use of bootstrap analyses, net benefit curves and CEACs. Added to two additional sensitivity assessments: intention-to-treat analysis and per-protocol analysis.	Total costs per patient in the CBT group (€1847) were lower than for patients who received RPT (€3664) or TAU (€3124). There was a higher quality of life in patients who received CBT. From a complete case analysis approach (base case), point estimates of cost-effectiveness ratios resulted in the CBT group being more advantageous in all comparisons performed, using both QALYs and EQ-VAS as outcomes.	Due to lower costs, CBT is the most cost-effective treatment for adult patients with FM. Thus, group CBT as a stand-alone intervention is cost-effective compared to FDA-recommended medications and usual care. Therefore, CBT programs in a group format should be expanded for patients with FM within the scope of public health care provision.
Luciano et al. [27]	Analysis of the efficiency of group acceptance and commitment therapy for fibromyalgia.	156 patients considered for inclusion were aged 18 to 65 years in a 6-month controlled trial in 3 conditions (using a computer-generated randomization list): group ACT (GACT), recommended pharmacological treatment (RPT), or wait list (WL). The FIQ was used to assess functional status.	Treatment with pregabalin (300–600 mg/d) was administered to FM patients. In addition, patients who fulfilled the criteria for major depression also received duloxetine (60–120 mg/d).		Group-based form of ACT was statistically superior to both recommended pharmacological treatment and wait list immediately after treatment, and improvements were maintained at 6 months in most cases.
Arnold et al. [28]	To evaluate, in patients with fibromyalgia and comorbid depression, the efficacy and safety of pregabalin, with concomitant use of antidepressant medication.	197 patients randomized, placebo-controlled, double-blind, crossover study.	Use of pregablin optimized to 300 or 450 mg per day.	There was a reduction in the level of pain with the use of pregabalin, significantly improving the scores on the Hospital Anxiety and Depression Scale and the total score of the Fibromyalgia Impact Questionnaire.	Pregabalin, compared to placebo, showed relief from FM pain and other symptoms in patients using antidepressant medications for comorbid depression.
Leombruni et al. [29]	Compare the effects of duloxetine and acetyl—L—carnitine on pain, depression, anxiety and well-being in patients with fibromyalgia	In this study, 65 female patients with FMS (fibromyalgia syndrome) were randomized to receive duloxetine or acetyl L-carnitine.	Use of duloxetine 60 mg/day or acetyl L-carnitine 1500 mg/day (500 mg three times a day) by female patients diagnosed by a rheumatologist.	The study showed that the two treatment links led to a general clinical improvement, with attenuation of pain and depressive symptoms. However, there was no significant reduction in anxiety. Both medications improved the physical component, but only duloxetine brought psychological improvement, in relation to increased quality of life.	In conclusion, duloxetine and acetyl-L-carnitine are effective in improving pain and depressive symptoms and the quality of life of patients with FMS.
Murakami et al. [30]	To evaluate the efficacy and safety of duloxetine in Japanese patients with fibromyalgia.	A double-blind, phase III study that randomized 393 patients to treatment with duloxetine 60 mg or placebo once daily for 14 weeks was carried out in Japan.	Use of duloxetine 60 mg or placebo once daily for 14 weeks in patients whose Brief Pain Inventory (BPI) pain score was ≥4 and who met the 1990 American College of Rheumatology criteria for fibromyalgia and whose mean score was pain.	From mixed-effects model repeated measures (MMRM) analysis, there were no differences between duloxetine and placebo with regard to change in mean BPI pain scores. Based on last observation carried forward (LOCF) analysis of covariance, a statistically significant improvement in change in mean BPI pain scores at week 14 was observed for patients treated with duloxetine compared to placebo. Treatment with duloxetine had a more satisfactory result in almost all secondary and post hoc analyses. The treatment was generally well tolerated. Drowsiness, nausea, and constipation were the most common treatment-emergent adverse events in the duloxetine group. Discontinuation rates due to treatment-emergent adverse events were similar in both groups.	Although the MMRM analysis did not demonstrate superiority of duloxetine over placebo, treatment with duloxetine was associated with satisfactory results in secondary and post hoc analyzes of mean change in mean BPI pain score and most secondary outcomes, including analgesia and quality of life. Treatment with duloxetine was safe and well tolerated. Thus, duloxetine may be associated with improvements in pain relief and quality of life in Japanese patients with fibromyalgia.
Gilron et al. [31]	This trial compares a pregabalin–duloxetine combination to each monotherapy for the management of fibromyalgia.	41 participants randomized, double-blind, 4-period crossover design, participants received maximally tolerated doses of placebo, pregabalin, duloxetine, and pregabalin–duloxetine combination—for 6 weeks.	Target daily dosage of 450 mg of pregabalin (maximum 3 capsules twice daily) and 120 mg of duloxetine (maximum 4 capsules daily).	Results of this trial demonstrate superiority of a pregabalin–duloxetine combination to pregabalin monotherapy; however, trends favouring combination over duloxetine monotherapy failed to reach significance. Although combination was not statistically superior to duloxetine for the primary outcome, this trial did demonstrate substantial superiority of combination to duloxetine monotherapy, as well as pregabalin, for global pain relief, functional improvement (Fibromyalgia Impact Questionnaire), and overall quality of life.	In conclusion, the results of this trial suggest that combining pregabalin with duloxetine can safely improve outcomes in fibromyalgia, including pain relief, physical function, and overall quality of life. These benefits may come with a risk of increased drowsiness.
Ramzy [32]	To investigate the effect of the combined use of pregabalin and paroxetine in the treatment of fibromyalgia.	75 female patients with fibromyalgia were randomized to receive different drugs. Individuals were evaluated for six months, every two months on scores on the Sompathic Symptom Scale-8 (SSS-8) and the Center for Epidemiological Studies Depression Scale (CESDS).	Patients were randomized to receive paroxetine (25 mg per day), venlafaxine (75 mg/day), or amitriptyline (25 mg per day).	There were improvements in SSS-8 and CESDS scores, improved medication tolerability, quality of life and sleep, and fewer cases of dry mouth and high blood pressure	The study concluded that the combined use of pregabalin with paroxetine is an effective method to improve patients’ quality of life by reducing fibromyalgia symptoms.
Murakami et al. [33]	To evaluate, in Japanese patients with fibromyalgia, the long-term effects of duloxetine 60 mg	A randomized, placebo-controlled, phase III study. The primary outcome was to evaluate the frequency of adverse events (AEs) and adverse drug reactions (ADRs)	149 patients in this study received duloxetine at a dose of 20 mg for one week, followed the next week by 40 mg and finally 60 mg for 48 weeks.	There was improvement at all time points compared to baseline (mean ± standard deviation change at Week 50 was −1.31 ± 1.70) in relation to the Brief Pain Inventory pain score.	The study concluded that long-term administration of duloxetine was safe and effective in treating Japanese patients with fibromyalgia.
Ghavidel-Parsa et al. [34]	Compare the effectiveness of one-month use of pregabalin and duloxetine in patients with FM	In this open randomized clinical study, patients with FM, diagnosed by a rheumatologist based on the 2016 American College of Rheumatology (ACR). Data from the primary outcome (Disease Invalidation Inventory (3*I)) and the secondary outcome (Inventory of Beck Depression-II (BDI-II), Generalized Pain Index (WPI), and Polysymptomatic Stress Scale (PSD)) were compared before and after treatment.	81 patients were randomized to treatment with duloxetine or pregabalin. In the treatment arm that used duloxetine, the drug was used at a dose of 30 mg for the first week and 60 mg for the last three weeks. In the research arm with the use of pregabalin, in the first week, 75 mg was administered and, in the other three weeks, 150 mg.	The study showed that duloxetine and pregabalin were effective in significantly improving WPI, BDI-II and PSD scores. However, there were no differences between the groups. There was also no significant change in the 3*I score.	Therefore, the research showed that, regardless of the medication, there was no effect on social pain in short-term pharmacological treatment.
Bruti et al. [35]	To evaluate the additional pain alleviation effect of Okada Purifying Therapy (OPT) when used concomitantly with duloxetine in patients with FM and Temporomandibular Disorders (TMDs).	The final sample in the research was 31 patients who were randomly divided between group 1 (who remained only on duloxetine) and group 2 (who underwent treatment with OPT)	Group 2 began with the administration of 30 mg of duloxetine for the first week and was then treated with duloxetine at 60 mg for seven weeks, adding two OPT sessions per week, for a total of 16 sessions. Group 1 remained on only duloxetine doses, the same as group 2.	No significant differences were found between the two groups 7 patients in group 1 did not complete the study due to the adverse effects of duloxetine, while 5 patients in group 2 did not complete the study due to not respecting the OPT session time.	OPT in conjunction with traditional treatment has not been shown to provide extra benefits for patients with TMD and FMS, but it appears to be related to improved pharmacological tolerability of conventional medication.
Salaffi et al. [36]	To comparatively analyze the effects of duloxetine (DLX) and pregabalin (PGB) in relation to palmitoylethanolamine (PEA) acetyl-L-carnitine (ALC) in patients with FM.	In this controlled and randomized study, 142 patients with FM after three months of stable treatment with DLX and PGB were divided into groups, group 1 to follow the treatment and group 2 to start using PEA in combination with ALC.	Initial use of 30 mg of duloxetine with 75 mg of pregabalin daily, followed by the use of 60 mg of DLX combined with 150 mg of PGB. After randomization, the control group (group 1) continued treatment without changes. Group 2 used 600 mg of PEA with 500 mg of ALC.	The AUC (area under the curve) values of the Widespread Pain Index (WPI) scale and FIQR scores decreased in group 2, compared to group 1.	The study demonstrated that there was efficacy in adding PEA and ALC to the treatment with pregabalin and duloxetine already carried out with patients with FM.
Ablin et al. [37]	To investigate, in comparison with pharmacological intervention, the effectiveness of Hyperbaric Oxygen Therapy (HBOT) in patients with FM related to brain trauma (TBI).	In this study, 64 patients with fibromyalgia related to TBI were randomized to HBOT or drug treatment (duloxetine or pregabalin).	Use of duloxetine with an initial dose of 30 mg or use of pregabalin with an initial dose of 75 mg. Compared to the HBO protocol, which comprised 60 sessions of 90 min, with 100% oxygen breathed through a mask.	The results showed that there was a decrease in pain intensity in patients who used HBOT treatment. Furthermore, there was also an improvement related to quality of life and an increase in pain thresholds in this group, compared to the group that uses pharmacological treatment.	The study concludes that Hyperbaric Oxygen Therapy, in relation to pharmacological treatment, proved to be more effective in alleviating pain and increasing quality of life in patients with fibromyalgia related to brain trauma.
Bidari et al. [38]	Compare the safety and efficacy of duloxetine and pregabalin.	A randomized clinical trial that administered duloxetine or pregabalin to women diagnosed with FM.	Use of duloxetine 30–60 mg compared to the use of pregabalin 75–150 mg, in female patients with fibromyalgia, between 18 and 65 years of age.	In all scales, only the WPI score showed a statistical difference between the two treatments, favoring duloxetine. However, this drug also had a higher dropout rate and a greater cumulative effect of nausea compared to pregabalin.	It is concluded, from this study, that there was greater effectiveness of treatment with duloxetine in relation to pregabalin.
Upadhyaya et al. [39]	To evaluate the efficacy and safety of 30 mg duloxetine for the treatment of juvenile fibromyalgia compared to placebo.	A phase 3b clinical trial, multicenter that randomized 184 patients to duloxetine with an initial dose of 30 mg. 91 received duloxetine and 93 placebo.	Use of duloxetine in patients who were between 13 and 17 years old, had fibromyalgia and had a score above or equal to 4 on the 24 h average pain intensity scale of the Brief Pain Inventory.	It was found that the intensity of pain, in the primary measurement, was not statically different. However, for secondary efficacy endpoints, more patients treated with duloxetine had statistically improved compared to those treated with placebo. There was a decrease of ≥30% and ≥50% in the average pain intensity of the BPI and improvement in general activity related to the same scale.	It is concluded that, in the primary outcome, there was no improvement in the 24 h BPI pain classification with duloxetine, compared to placebo in juvenile fibromyalgia patients. However, significantly more patients had a decrease in pain intensity when receiving duloxetine compared to placebo. Additionally, there were no new concerns regarding adverse effects and safety concerns related to the drug.

## Data Availability

No new data were created or analyzed in this study.

## References

[1] Hawkins R.A. (2013). Fibromyalgia: A Clinical Update. J. Osteopath. Med..

[2] Sarzi-Puttini P., Giorgi V., Atzeni F., Gorla R., Kosek E., Choy E.H., Bazzichi L., Häuser W., Ablin J.N., Aloush V. (2021). Fibromyalgia Position Paper. Clin. Exp. Rheumatol..

[3] Paredes S., Cantillo S., Candido K.D., Knezevic N.N. (2019). An Association of Serotonin with Pain Disorders and Its Modulation by Estrogens. Int. J. Mol. Sci..

[4] Martínez-Lavín M. (2021). Fibromyalgia in Women: Somatisation or Stress-Evoked, Sex-Dimorphic Neuropathic Pain?. Clin. Exp. Rheumatol..

[5] Bennett R.M., Jones J., Turk D.C., Russell I.J., Matallana L. (2007). An Internet Survey of 2596 People with Fibromyalgia. BMC Musculoskelet. Disord..

[6] Wolfe F., Clauw D.J., Fitzcharles M., Goldenberg D.L., Katz R.S., Mease P., Russell A.S., Russell I.J., Winfield J.B., Yunus M.B. (2010). The American College of Rheumatology Preliminary Diagnostic Criteria for Fibromyalgia and Measurement of Symptom Severity. Arthritis Care Res..

[7] Lawson K. (2017). Emerging Pharmacological Strategies for the Treatment of Fibromyalgia. World J. Pharmacol..

[8] Bazzichi L., Giacomelli C., Consensi A., Giorgi V., Batticciotto A., Di Franco M., Sarzi-Puttini P. (2020). One Year in Review 2020: Fibromyalgia. Clin. Exp. Rheumatol..

[9] De Oliveira Júnior J.O., de Almeida M.B. (2018). The Current Treatment of Fibromyalgia. Braz. J. Pain.

[10] Maffei M.E. (2020). Fibromyalgia: Recent Advances in Diagnosis, Classification, Pharmacotherapy and Alternative Remedies. Int. J. Mol. Sci..

[11] Brietzke A.P., Antunes L.C., Carvalho F., Elkifury J., Gasparin A., Sanches P.R.S., da Silva Junior D.P., Dussán-Sarria J.A., Souza A., da Silva Torres I.L. (2019). Potency of Descending Pain Modulatory System Is Linked with Peripheral Sensory Dysfunction in Fibromyalgia. Medicine.

[12] Bradley L.A. (2009). Pathophysiology of Fibromyalgia. Am. J. Med..

[13] Teleanu R.I., Niculescu A.-G., Roza E., Vladâcenco O., Grumezescu A.M., Teleanu D.M. (2022). Neurotransmitters—Key Factors in Neurological and Neurodegenerative Disorders of the Central Nervous System. Int. J. Mol. Sci..

[14] Macian N., Pereira B., Shinjo C., Dubray C., Pickering G. (2015). Fibromyalgia, Milnacipran and Experimental Pain Modulation: Study Protocol for a Double Blind Randomized Controlled Trial. Trials.

[15] Mease P.J. (2009). Further Strategies for Treating Fibromyalgia: The Role of Serotonin and Norepinephrine Reuptake Inhibitors. Am. J. Med..

[16] Jensen K.B., Petzke F., Carville S., Choy E., Fransson P., Gracely R.H., Vitton O., Marcus H., Williams S.C.R., Ingvar M. (2014). Segregating the Cerebral Mechanisms of Antidepressants and Placebo in Fibromyalgia. J. Pain.

[17] Sansone R.A., Sansone L.A. (2014). Serotonin Norepinephrine Reuptake Inhibitors:A Pharmacological Comparison. Innov. Clin. Neurosci..

[18] Mika J., Zychowska M., Makuch W., Rojewska E., Przewlocka B. (2013). Neuronal and Immunological Basis of Action of Antidepressants in Chronic Pain—Clinical and Experimental Studies. Pharmacol. Reports.

[19] Macfarlane G.J., Kronisch C., Dean L.E., Atzeni F., Häuser W., Flub E., Choy E., Kosek E., Amris K., Branco J. (2017). EULAR Revised Recommendations for the Management of Fibromyalgia. Ann. Rheum. Dis..

[20] Welsch P., Üçeyler N., Klose P., Walitt B., Häuser W. (2018). Serotonin and noradrenaline reuptake inhibitors (SNRIs) for fibromyalgia. Cochrane Database Syst. Rev..

[21] Casale R., Sarzi-Puttini P., Botto R., Alciati A., Batticciotto A., Marotto D., Torta R. (2019). Fibromyalgia and the Concept of Resilience. Clin. Exp. Rheumatol..

[22] Gaynor P.J., Liu P., Weller M.A., Wohlreich M.M. (2013). Comparison of Safety Outcomes among Caucasian, Hispanic, Black, and Asian Patients in Duloxetine Studies of Chronic Painful Conditions. Curr. Med. Res. Opin..

[23] Schmidt-Wilcke T., Ichesco E., Hampson J.P., Kairys A., Peltier S., Harte S., Clauw D.J., Harris R.E. (2014). Resting State Connectivity Correlates with Drug and Placebo Response in Fibromyalgia Patients. NeuroImage Clin..

[24] Moore R.A., Cai N., Skljarevski V., Tölle T.R. (2014). Duloxetine Use in Chronic Painful Conditions—Individual Patient Data Responder Analysis. Eur. J. Pain.

[25] Lipkovich I.A., Choy E.H., Van Wambeke P., Deberdt W., Sagman D. (2014). Typology of Patients with Fibromyalgia: Cluster Analysis of Duloxetine Study Patients. BMC Musculoskelet. Disord..

[26] Luciano J.V., D’Amico F., Cerdà-Lafont M., Peñarrubia-María M.T., Knapp M., Cuesta-Vargas A.I., Serrano-Blanco A., García-Campayo J. (2014). Cost-Utility of Cognitive Behavioral Therapy versus U.S. Food and Drug Administration Recommended Drugs and Usual Care in the Treatment of Patients with Fibromyalgia: An Economic Evaluation alongside a 6-Month Randomized Controlled Trial. Arthritis Res. Ther..

[27] Luciano J.V., Guallar J.A., Aguado J., López-del-Hoyo Y., Olivan B., Magallón R., Alda M., Serrano-Blanco A., Gili M., Garcia-Campayo J. (2014). Effectiveness of Group Acceptance and Commitment Therapy for Fibromyalgia: A 6-Month Randomized Controlled Trial (EFFIGACT Study). Pain.

[28] Arnold L.M., Sarzi-Puttini P., Arsenault P., Khan T., Bhadra Brown P., Clair A., Scavone J.M., Driscoll J., Landen J., Pauer L. (2015). Efficacy and Safety of Pregabalin in Patients with Fibromyalgia and Comorbid Depression Taking Concurrent Antidepressant Medication: A Randomized, Placebo-Controlled Study. J. Rheumatol..

[29] Leombruni P., Miniotti M., Colonna F., Sica C., Castelli L., Bruzzone M., Parisi S., Fusaro E., Sarzi-Puttini P., Atzeni F. (2015). A Randomised Controlled Trial Comparing Duloxetine and Acetyl L-Carnitine in Fibromyalgic Patients: Preliminary Data. Clin. Exp. Rheumatol..

[30] Murakami M., Osada K., Mizuno H., Ochiai T., Alev L., Nishioka K. (2015). A Randomized, Double-Blind, Placebo-Controlled Phase III Trial of Duloxetine in Japanese Fibromyalgia Patients. Arthritis Res. Ther..

[31] Gilron I., Chaparro L.E., Tu D., Holden R.R., Milev R., Towheed T., DuMerton-Shore D., Walker S. (2016). Combination of Pregabalin with Duloxetine for Fibromyalgia: A Randomized Controlled Trial. Pain.

[32] Ramzy E.A. (2017). Comparative Efficacy of Newer Antidepressants in Combination with Pregabalin for Fibromyalgia Syndrome: A Controlled, Randomized Study. Pain Pract..

[33] Murakami M., Osada K., Ichibayashi H., Mizuno H., Ochiai T., Ishida M., Alev L., Nishioka K. (2017). An Open-Label, Long-Term, Phase III Extension Trial of Duloxetine in Japanese Patients with Fibromyalgia. Mod. Rheumatol..

[34] Ghavidel-Parsa B., Bidari A., Rahimi A., Gharibpoor F., Khosousi M.-J. (2022). No Effect of Approved Fibromyalgia Drugs on the Social Pain (Invalidation) Contrary to Physical Pain: An Open-Label Short-Term Randomized Clinical Trial. Clin. Rheumatol..

[35] Bruti G., Atencio M.R., D’Urso A., Di Giacomo P., Di Paolo C. (2021). Okada Purifying Therapy in Combination with Duloxetine vs. Duloxetine Alone in Patients with TMD and Fibromyalgia: A Randomized Clinical Study. J. Complement. Integr. Med..

[36] Salaffi F., Farah S., Sarzi-Puttini P., Di Carlo M. (2023). Palmitoylethanolamide and Acetyl-L-Carnitine Act Synergistically with Duloxetine and Pregabalin in Fibromyalgia: Results of a Randomised Controlled Study. Clin. Exp. Rheumatol..

[37] Ablin J.N., Lang E., Catalogna M., Aloush V., Hadanny A., Doenyas-Barak K., Finci S., Polak N., Fishlev G., Korin C. (2023). Hyperbaric Oxygen Therapy Compared to Pharmacological Intervention in Fibromyalgia Patients Following Traumatic Brain Injury: A Randomized, Controlled Trial. PLoS ONE.

[38] Bidari A., Moazen-Zadeh E., Ghavidel-Parsa B., Rahmani S., Hosseini S., Hassankhani A. (2019). Comparing Duloxetine and Pregabalin for Treatment of Pain and Depression in Women with Fibromyalgia: An Open-Label Randomized Clinical Trial. DARU J. Pharm. Sci..

[39] Upadhyaya H.P., Arnold L.M., Alaka K., Qiao M., Williams D., Mehta R. (2019). Efficacy and Safety of Duloxetine versus Placebo in Adolescents with Juvenile Fibromyalgia: Results from a Randomized Controlled Trial. Pediatr. Rheumatol..

[40] García Rodríguez D.F., Abud Mendoza C. (2020). Fisiopatología de La Fibromialgia. Reumatol. Clín..

[41] Giorgi V., Sirotti S., Romano M.E., Marotto D., Ablin J.N., Salaffi F., Sarzi-Puttini P. (2022). Fibromyalgia: One Year in Review 2022. Clin. Exp. Rheumatol..

[42] Alzabibi M.A., Shibani M., Alsuliman T., Ismail H., Alasaad S., Torbey A., Altorkmani A., Sawaf B., Ayoub R., Khalayli N. (2022). Fibromyalgia: Epidemiology and Risk Factors, a Population-Based Case-Control Study in Damascus, Syria. BMC Rheumatol..

[43] Siracusa R., Di Paola R., Cuzzocrea S., Impellizzeri D. (2021). Fibromyalgia: Pathogenesis, Mechanisms, Diagnosis and Treatment Options Update. Int. J. Mol. Sci..

[44] Ferreira-dos-santos G., Sousa D.C. (2018). Análise Da Revisão Cochrane: Pregabalina No Tratamento Da Dor Crónica Moderada a Grave Em Adultos Com Fibromialgia. Cochrane Database Syst Rev. 2016; 9: CD011790 e 2016; 4: CD009002. Acta Med. Portuguesa.

[45] Zhang X., Xu H., Zhang Z., Li Y., Pauer L., Liao S., Zhang F. (2021). Efficacy and Safety of Pregabalin for Fibromyalgia in a Population of Chinese Subjects. J. Pain Res..

[46] Smith H.S., Bracken D., Smith J.M. (2010). Duloxetine: A Review of Its Safety and Efficacy in the Management of Fibromyalgia Syndrome. J. Cent. Nerv. Syst. Dis..

[47] Kashikar-Zuck S., Ting T.V. (2014). Juvenile Fibromyalgia: Current Status of Research and Future Developments. Nat. Rev. Rheumatol..

[48] Häuser W., Petzke F., Sommer C. (2010). Comparative Efficacy and Harms of Duloxetine, Milnacipran, and Pregabalin in Fibromyalgia Syndrome. J. Pain.

[49] Farag H.M., Yunusa I., Goswami H., Sultan I., Doucette J.A., Eguale T. (2022). Comparison of Amitriptyline and US Food and Drug Administration-Approved Treatments for Fibromyalgia: A Systematic Review and Network Meta-Analysis. JAMA Netw. Open.

[50] Derry S., Cording M., Wiffen P.J., Law S., Phillips T., Moore R.A. (2016). Pregabalin for Pain in Fibromyalgia in Adults. Cochrane Database Syst. Rev..

[51] Üçeyler N., Häuser W., Sommer C. (2008). A Systematic Review on the Effectiveness of Treatment with Antidepressants in Fibromyalgia Syndrome. Arthritis Care Res..

[52] Sultan A., Gaskell H., Derry S., Andrew R.A. (2008). Duloxetine for Painful Diabetic Neuropathy and Fibromyalgia Pain: Systematic Review of Randomised Trials. BMC Neurol..

[53] Rodrigues-Amorim D., Olivares J.M., Spuch C., Rivera-Baltanás T. (2020). A Systematic Review of Efficacy, Safety, and Tolerability of Duloxetine. Front. Psychiatry.

[54] Konno S., Oda N., Ochiai T., Alev L. (2016). Randomized, Double-Blind, Placebo-Controlled Phase III Trial of Duloxetine Monotherapy in Japanese Patients With Chronic Low Back Pain. Spine.

[55] Derry S., Phillips T., Moore R.A., Wiffen P.J. (2015). Milnacipran for Neuropathic Pain in Adults. Cochrane Database Syst. Rev..

[56] Derry S., Gill D., Phillips T., Moore R.A. (2012). Milnacipran for Neuropathic Pain and Fibromyalgia in Adults. Cochrane Database Syst. Rev..

[57] Cording M., Derry S., Phillips T., Moore R.A., Wiffen P.J. (2015). Milnacipran for Pain in Fibromyalgia in Adults. Cochrane Database Syst. Rev..

[58] Nielsen F.Å., Balslev D., Hansen L.K. (2005). Mining the Posterior Cingulate: Segregation between Memory and Pain Components. Neuroimage.

[59] Arnold L.M., Bateman L., Palmer R.H., Lin Y. (2015). Preliminary Experience Using Milnacipran in Patients with Juvenile Fibromyalgia: Lessons from a Clinical Trial Program. Pediatr. Rheumatol..

[60] Zernikow B., Gerhold K., Bürk G., Häuser W., Hinze C.H., Hospach T., Illhardt A., Mönkemöller K., Richter M., Schnöbel-Müller E. (2012). Definition, Diagnostik Und Therapie von Chronischen Schmerzen in Mehreren Körperregionen Und Des Sogenannten Fibromyalgiesyndroms Bei Kindern Und Jugendlichen. Der Schmerz.

[61] Wu Y., Guo X., Zhang J. (2025). Calcium Channel α_2_δ Ligands Mirogabalin, Pregabalin, and Gabapentin: Advancements in Diabetic Peripheral Neuropathic Pain Therapeutics. Pain Ther..

[62] Malik A., Brown Z., Ryder A., Balayan V., Jameson L., Vu P., Al-Jumah R., Hasoon J. (2025). Serotonin and Norepinephrine Reuptake Inhibitors (SNRIs) for Treatment of Fibromyalgia: A Comprehensive Clinical Review. Psychopharmacol. Bull..

[63] Goldstein D.J., Lu Y., Detke M.J., Hudson J., Iyengar S., Demitrack M.A. (2004). Effects of duloxetine on painful physical symptoms associated with depression. Psychosomatics.

[64] Ilari S., Passacatini L.C., Malafoglia V., Oppedisano F., Maiuolo J., Gliozzi M., Palma E., Tomino C., Fini M., Raffaeli W. (2022). Tantali Fibromyalgic Supplicium: Is There Any Relief with the Antidepressant Employment? A Systematic Review. Pharmacol. Res..

[65] Lunn M.P.T., Hughes R.A.C., Wiffen P.J. (2014). Duloxetine for Treating Painful Neuropathy, Chronic Pain or Fibromyalgia. Cochrane Database Syst. Rev..

[66] Arnold L.M., Lu Y., Crofford L.J., Wohlreich M., Detke M.J., Iyengar S., Goldstein D.J. (2004). A Double-blind, Multicenter Trial Comparing Duloxetine with Placebo in the Treatment of Fibromyalgia Patients with or without Major Depressive Disorder. Arthritis Rheum..

[67] Arnold L.M., Rosen A., Pritchett Y.L., D’Souza D.N., Goldstein D.J., Iyengar S., Wernicke J.F. (2005). A Randomized, Double-Blind, Placebo-Controlled Trial of Duloxetine in the Treatment of Women with Fibromyalgia with or without Major Depressive Disorder. Pain.

[68] Nose M., Cipriani A., Furukawa T.A., Omori I.M., Churchill R., McGuire H.F., Barbui C. (2012). Duloxetine versus Other Anti-Depressive Agents for Depression. Cochrane Database Syst. Rev..

[69] Koechlin H., Kharko A., Probst T., Pradela J., Buechi S., Locher C. (2021). Placebo Responses and Their Clinical Implications in Fibromyalgia: A Meta-Analysis Using SSRI and SNRI Trials. Front. Pain Res..

[70] Abdel Fattah Y.H., Elnemr R. (2020). Efficacy of Pregabalin as a Monotherapy versus Combined Pregabalin and Milnacipran in the Management of Fibromyalgia. Int. J. Rheum. Dis..

[71] Thorpe J., Gilron I., Shum B., Moore R.A., Wiffen P.J. (2013). Combination Pharmacotherapy for the Treatment of Fibromyalgia. Cochrane Database Syst. Rev..

