# Correction: Lago et al. Serotonin–Norepinephrine Reuptake Inhibitors in Fibromyalgia Management: An Integrative Literature Review of Clinical Evidence. *Clin. Pract.* 2026, *16*, 68

**DOI:** 10.3390/clinpract16050084

**Published:** 2026-04-29

**Authors:** Isabella Oliveira do Lago, Bruna Moura Medina Diniz, Daniela Vieira Buchaim, Rogerio Leone Buchaim

**Affiliations:** 1 Bauru School of Medicine, University of Sao Paulo (FMBRU/USP), Bauru 17012-901, Brazil; isabella.lago@usp.br; 2 Ribeirão Preto School of Medicine, University of Sao Paulo (FMRP/USP), Ribeirão Preto 14049-900, Brazil; brunamedina@usp.br; 3 Medical School, University Center of Adamantina (FAI), Adamantina 17800-000, Brazil; danibuchaim@alumni.usp.br; 4 Center for the Study of Venoms and Venomous Animals (CEVAP), Sao Paulo State University (UNESP), Botucatu 18610-307, Brazil; 5 Postgraduate Department, Dentistry School, Faculty of the Midwest Paulista (FACOP), Piratininga 17499-010, Brazil; 6 Graduate Program in Anatomy of Domestic and Wild Animals, Faculty of Veterinary Medicine and Animal Science, University of Sao Paulo (FMVZ/USP), Sao Paulo 05508-270, Brazil; 7 Department of Biological Sciences, Bauru School of Dentistry (FOB/USP), University of Sao Paulo, Bauru 17012-901, Brazil

## 1. Text Correction

In the original publication [1], the first two paragraphs of the “1. Introduction” section are repetitive. The content of these two paragraphs should be changed to:

Fibromyalgia (FM) is a disorder of central pain processing and affects 2–4% of the global population, predominantly females aged 20–55 years, characterized by widespread musculoskeletal pain (prevalence 63–90%), chronic fatigue (81%), non-restorative sleep, depression, and muscle stiffness per ACR 2010/2016 criteria. In addition, the incomplete understanding of the pathophysiology of FM, whose clinical manifestations have no known cause but rather imply its multifactorial nature, results in the absence of objective diagnostic tests and specific treatments [1–6].

## 2. Errors in Table

In the original publication [1], in Table 1, the sentence “Some studies used multiple therapies, leading to quantitative replication in this analysis” is missing. The correct Table 1 is as follows:

The authors state that the scientific conclusions are unaffected. This correction was approved by the Academic Editor. The original publication has also been updated.

## Figures and Tables

**Table 1:** Characteristics of the included studies according to type of inhibitor, SNRI application methodology, and restrictive inclusion criteria.

Dimension	Category/Description	Number of Studies	Notes
Type of pain-related inhibitor	Duloxetine	16	Studies using Duloxetine as the main neurotransmitter inhibitor associated with pain.
	Milnacipran	2	Some studies used multiple therapies, leading to quantitative replication in this analysis.
	Venlafaxine	2	
	Desvenlafaxine	1	
	SSRIs (Paroxetine highlighted)	2	Selective serotonin reuptake inhibitors.
SNRI application methodology	SNRIs vs. placebo	8	Some studies used multiple methods, leading to quantitative replication in this analysis.
	SNRIs vs. pregabalin	3	
	SNRIs + pregabalin (combination)	4	
	SNRIs vs. other drugs (antidepressants, palmitoylethanolamide, acetyl-L-carnitine)	2	
	SNRIs vs. other therapies (Okada purifying therapy, HBOT)	2	HBOT: hyperbaric oxygen therapy.
	Gradual dose increase in SNRIs	2	
Restrictive inclusion criteria	Japanese participants only	3	Criteria limiting generalizability to the broader fibromyalgia population.
	Females only	3	
	Younger individuals	1	
	Prior conditions (brain trauma, depression, chronic pain, TMD)	4	TMD: temporomandibular disorder.

Due to fibromyalgia’s complexity, the selected studies focused on neurotransmitter inhibitors linked to pain. Duloxetine predominated among inhibitors. SNRI methodologies included comparisons with placebo, pregabalin, other drugs/therapies, and dose escalation (with some overlap). Restrictive criteria (e.g., ethnicity, sex, age, comorbidities) were common, limiting generalizability.

## References

[1] Lago I.O.d., Diniz B.M.M., Buchaim D.V., Buchaim R.L. (2026). Serotonin–Norepinephrine Reuptake Inhibitors in Fibromyalgia Management: An Integrative Literature Review of Clinical Evidence. Clin. Pract..

